# The Role of Electrostatic Binding Interfaces in the Performance of Bacterial Reaction Center Biophotoelectrodes

**DOI:** 10.1021/acssuschemeng.2c06769

**Published:** 2023-02-07

**Authors:** Milo R. van Moort, Michael R. Jones, Raoul N. Frese, Vincent M. Friebe

**Affiliations:** † Biophysics of Photosynthesis, Department of Physics and Astronomy, Faculty of Science, Vrije Universiteit Amsterdam, 1081 HV Amsterdam, The Netherlands; ‡ LaserLaB Amsterdam, Vrije Universiteit Amsterdam, 1081 HV Amsterdam, The Netherlands; § School of Biochemistry, Biomedical Sciences Building, University of Bristol, University Walk, Bristol BS8 1TD, United Kingdom; ∥ Campus Straubing for Biotechnology and Sustainability, Technical University of Munich, Uferstraße 53, 94315 Straubing, Germany

**Keywords:** biosolar cells, biophotovoltaics, biophotoelectrochemistry, reaction center, cytochrome *c*

## Abstract

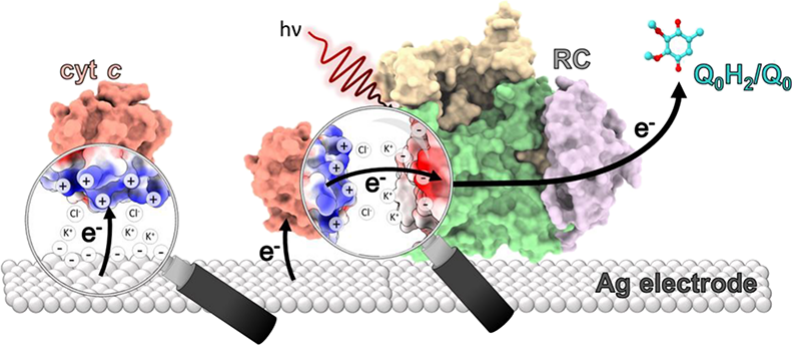

Photosynthetic reaction centers (RCs) efficiently capture and convert solar radiation into electrochemical energy. Accordingly, RCs have the potential as components in biophotovoltaics, biofuel cells, and biosensors. Recent biophotoelectrodes containing the RC from the bacterium *Rhodobacter sphaeroides* utilize a natural electron donor, horse heart cytochrome *c* (cyt *c*), as an electron transfer mediator with the electrode. In this system, electrostatic interfaces largely control the protein–electrode and protein–protein interactions necessary for electron transfer. However, recent studies have revealed kinetic bottlenecks in cyt-mediated electron transfer that limit biohybrid photoelectrode efficiency. Here, we seek to understand how changing protein–protein and protein–electrode interactions influence RC turnover and biophotoelectrode efficiency. The RC–cyt *c* binding interaction was modified by substituting interfacial RC amino acids. Substitutions Asn-M188 to Asp and Gln-L264 to Glu, which are known to produce a higher cyt-binding affinity, led to a decrease in the RC turnover frequency (TOF) at the electrode, suggesting that a decrease in cyt *c* dissociation was rate-limiting in these RC variants. Conversely, an Asp-M88 to Lys substitution producing a lower binding affinity had little effect on the RC TOF, suggesting that a decrease in the cyt *c* association rate was not a rate-limiting factor. Modulating the electrode surface with a self-assembled monolayer that oriented the cyt *c* to face the electrode did not affect the RC TOF, suggesting that the orientation of cyt *c* was also not a rate-limiting factor. Changing the ionic strength of the electrolyte solution had the most potent impact on the RC TOF, indicating that cyt *c* mobility was important for effective electron donation to the photo-oxidized RC. An ultimate limitation for the RC TOF was that cyt *c* desorbed from the electrode at ionic strengths above 120 mM, diluting its local concentration near the electrode-adsorbed RCs and resulting in poor biophotoelectrode performance. These findings will guide further tuning of these interfaces for improved performance.

## Abstract 2

The electrostatic interfaces between sustainable photoproteins and electrodes play a major role in the efficient conversion of solar into electrochemical energy.

## Introduction

The high efficiency and adaptability of natural photosynthetic reaction center (RC) proteins underpin their potential as sustainable components in biohybrid photoelectrodes for solar energy conversion^1^ and applications such as biosensing.^2,3^ These intramembrane pigment proteins use light energy to separate charge across the photosynthetic membrane followed by external electron transfer that stabilizes the intraprotein charge separation and hence the energy conversion. A principal challenge in the development of biohybrid RC photoelectrodes is achieving an efficient transfer of electrons from the electrode following photochemical charge separation within the RC.^4^ The electrostatic interactions occurring between proteins and the electrode,^5^ and between adjacent proteins,^6^ form interfacial boundaries that may result in kinetic bottlenecks in the biohybrid electron transfer chain.^4^ Improvement of the performance of biophotoelectrodes requires a better understanding of the impact of these interfaces on electron transfer from the electrode.

**Figure 1:**
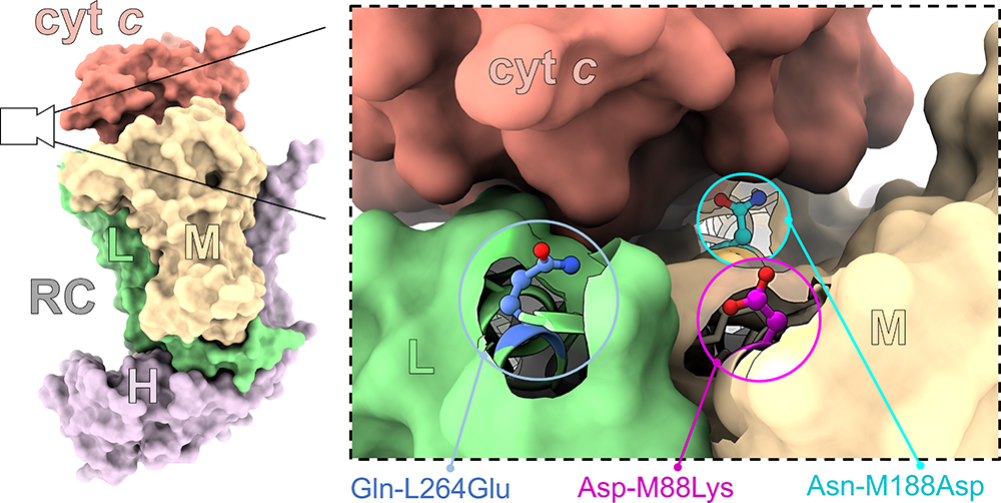
Reaction center structure and mutations. (Left) overview of the interaction complex between the *Rba. sphaeroides* RC and cyt *c*_2_, taken from an X-ray crystal structure of the cocomplex (Protein Data Bank ID: 1L9B^7^) and rendered using ChimeraX.^8^ The RC L-subunit (green), M-subunit (tan), and H-subunit (purple) are labeled. (Right) a zoomed-in view of the RC/cyt *c* binding interface depicting the location of the substituted residues Gln-L264 (blue carbons: changed to Glu), Asp-M88 (magenta carbons: changed to Lys), and Asn-M188 (cyan carbons: changed to Asp).

In the much used *Rhodobacter (Rba.) sphaeroides* RC (Figure 1), light-driven charge separation results in the oxidation of a pair of bacteriochlorophyll cofactors (P870) at one end of an intraprotein electron transfer chain and the reduction of a ubiquinone-10 (Q_B_) at the opposite end.^9^ At the oxidized terminus, charge separation is stabilized by reduction of P870^+^ by a small mobile mono-heme cytochrome *c*_2_ (cyt *c*_2_),^10^ which docks onto a site on the extramembrane surface of the RC adjacent to the buried P870^+^ (Figure 1). Donation of an electron resets P870^+^ for further charge separation, and oxidized cyt *c*_2_ then undocks to replenish its lost electron.^11^ A detailed description of all potential binding, unbinding, and electron transfer steps is given in Figure S1 and the associated text. Docking of cyt *c*_2_ to the RC is controlled by an electrostatic binding interface that has been extensively studied using a variety of methods, including protein engineering of the RC to strengthen or weaken cyt binding.^12−17^ Fitting of the rate of P870^+^ reduction requires a first-order electron transfer rate constant (*k*_1_) of ∼10^6^ s^–1^ that describes microsecond P870^+^ reduction in preformed RC–cyt *c*_2_ complexes and a slower, and therefore limiting, second-order electron transfer rate constant (*k*_2_) in the region of 10^9^ M^–1^ s^–1^ that describes the millisecond docking of cyt *c*_2_ to the RC.^18^ The protein–protein interaction surface involves an area with a predominantly negative surface potential on the surface of the RC and a complementary predominantly positively charged surface on the cyt *c*_2_ that enables initial binding and positions the heme for electron transfer.

As the *Rhodobacter* cyt *c*_2_ is similar to commercially available mitochondrial cyt *c*, the latter has been used as a convenient substitute in a range of *in vitro* studies,^19,20^ including the assembly of a variety of biohybrid photoelectrodes in which cyt *c* acts as an electron relay between the RC and the conductive substrate.^5,13,21−23^ In a number of aspects, the mechanism of cyt *c* → RC electron transfer at an electrode surface is different from that occurring *in vivo* or in experiments conducted in solution. First, to form an electron transfer relay with the RC, free cyt *c* has to adsorb onto the electrode in a manner determined by an electrode–cyt *c* binding equilibrium constant (*K*_E_).^21^ Second, to support a photocurrent over multiple RC turnovers, cyt *c* molecules have to remain largely confined to the plane of the electrode surface during fabrication and operation. That this occurs is supported by studies that have shown that the photocurrents remain stable for hours after free cyt *c* is removed from the electrolyte.^23−25^ Third, the orientation of the RC on the electrode may occlude cyt *c* access, restricting the rate of P870^+^ reduction.^26^ These differences result in RC electron transfer turnover frequencies (TOF) that are typically on the order of 10 to 150 e^–^ s^–1^.^21,24^ These are only a fraction of the maximal RC TOF of up to 2300 e^–^ s^–1^ that can be observed with purified proteins in solution.^20,27^

A recent study using spectroelectrochemistry has shown that a limiting factor for RC turnover on an electrode is a kinetic bottleneck associated with cyt *c-*mediated electron transfer,^4^ indicating a parameter that could be adjusted for better overall performance. In the present work, we characterized biophotoelectrode performance in response to modifications expected to impact the electrostatic interfaces between the RC and cyt *c* and cyt *c* and an electrode. Protein engineering was used to increase the affinity with which cyt *c* binds to the RC, the electrode–cyt *c* interface was modified by functionalizing the electrode using a negatively charged self-assembled monolayer (SAM), and the ionic strength of the electrolyte was systematically varied. The findings shed new light on how the electrostatic interface between cyt *c* and the RC influences turnover in a biohybrid electrode setting and how these protein complexes are configured on an electrode, providing information to aid future designs of more efficient biophotoelectrodes.

## Results

To investigate the role of the RC–cyt *c* electrostatic interactions on the biophotoelectrode activity, three variants of the wild-type (WT) RC were engineered with a single residue substitution in the predominantly anionic docking site for cyt *c*_2_ and cyt *c* (Figure 1). In mutation Asp-M88Lys, a negatively charged aspartic acid at position 88 of the RC M-polypeptide was replaced by a positively charged lysine. This substitution is known to result in an ∼200-fold lower RC–cyt *c* binding constant (*K*_B_ = 1/*K*_D_) and produce a decreased second-order rate constant (*k*_2_) for reduction of P870^+^ (Table 1).^6,18^ In mutations Asn-M188Asp and Gln-L264Glu, a neutral residue was replaced by a structurally similar negatively charged residue. These mutations are known to increase *K*_B_ and *k*_2_ (Table 1).^6,18^ Despite their contrasting effects on *k*_2_, which describes the rate of cyt *c* docking (Table 1), the three mutations have a minimal impact on *k*_1_.^18^

**Table 1:** Parameters Characterizing the Behavior of RC Turnover *In Vitro* and on an Electrode

sample	peak *J*_photo_^a^ (μA cm^–2^)	Γ_RC_^a^ (pmol cm^–2^)	max RC TOF^a^ (e^–^ s^–1^ RC^–1^)	*K*_PC_^a^ (μM)	*n*^a^ (a.u.)	*K*_D_^b^ (μM)	*k*_2_ ≈ *k*_ON_^b^(×10^9^ M^–1^ s^–1^)	*k*_OFF_^b^ (s^–1^)
WT	23 ± 4	80 ± 3	3 ± 0.6	3.6 ± 0.1	3.4 ± 0.2	0.30	1.7	1000
Asp-M88Lys	13 ± 2	51 ± 2	2.6 ± 0.4	2.7 ± 0.2	3.7 ± 0.5	55	0.2	22,000
Asn-M188Asp	6 ± 1	54 ± 6	1.2 ± 0.2	2.3 ± 0.1	3.0 ± 0.1	0.06	2.5	300
Gln-L264Glu	9.2 ± 1.7	52 ± 2	1.8 ± 0.4	2.9 ± 0.1	2.0 ± 0.1	0.01	3.0	60
WT SAM-Ag^R^	5.8 ± 1.2	25 ± 2	2.7 ± 0.5	2.8 ± 0.1	1.6 ± 0.1			

a Peak photocurrents at 20 μM cyt *c* (*J*_photo_), RC loadings (Γ_RC_), maximum RC turnover frequencies (TOF), and half-maximal photocurrent cyt *c* concentration (*K*_PC_) were determined as described in Materials and Methods. The parameter *n* is the cyt *c* electron transfer cooperativity of the Hill fit. All values are shown with their standard deviations (*n* = 3).

b Solution RC–cyt *c* dissociation constants (*K*_D_) and second-order electron transfer rate constants (*k*_2_) were derived from published data^18^ and have an experimental error of less than 15%. Rates of unbinding (*k*_OFF_) were calculated using *K*_D_ = *k*_ON_/*k*_OFF_ where it was assumed that *k*_2_ ≈ *k*_ON_ at a low ionic strength and a free cyt *c* concentration of 20 μM. First-order rate constants (*k*_1_) are excluded from Table 1 since they are on the order of μs, unaffected by mutagenesis^18^ and not rate-limiting.

**Figure 2:**
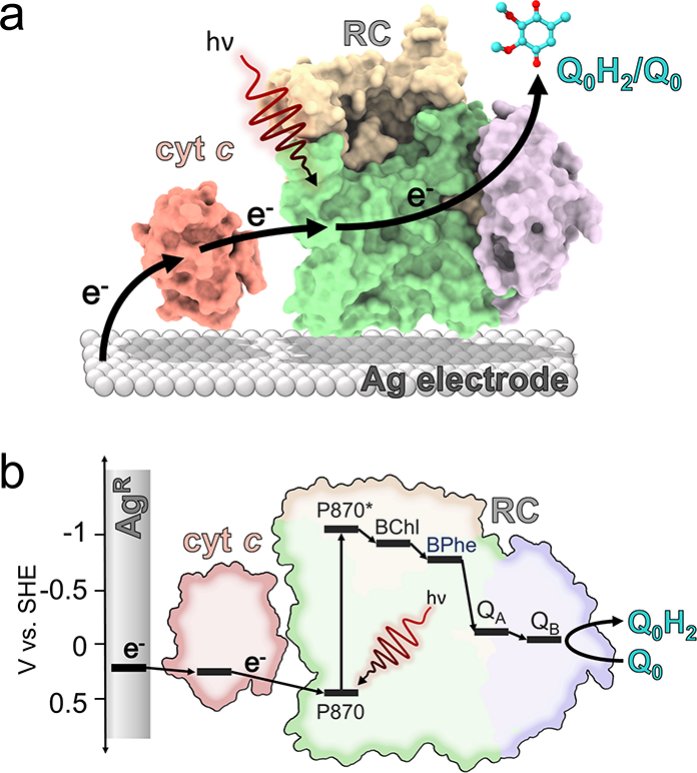
Biophotoelectrode configuration and mechanism. (a) Schematic depicting the composition and arrangement of the RC, cyt *c*, Q_0_, and the mesoporous silver electrode (Ag^R^). The mesoporous structure of the Ag^R^ is omitted for clarity. The electron transfer pathway is indicated by the black arrows. (b) Plot of the midpoint potentials of all components involved in the electron transfer pathway, including the bacteriochlorophyll pair (P870), sequential monomeric bacteriochlorophyll (BChl), bacteriopheophytin (BPhe), and ubiquinone (Q_A_ and Q_B_) electron carriers. The added water-soluble Q_0_ carries electrons to the Pt counter electrode (not shown).

**Figure 3:**
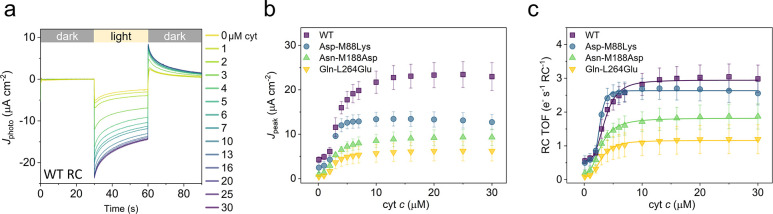
Dependence of electrode performance on cyt *c* concentration. (a) Averaged photocurrents from WT RCs at an increasing cyt *c* concentration. Error bars are omitted for clarity. The period of illumination is indicated by the yellow bar. (b) Peak cathodic photocurrents as a function of cyt *c* concentration for four bioelectrodes with different RCs. (c) RC TOF as a function of cyt *c* concentration (symbols) overlaid with a Hill equation fit (lines), which converged with an *R*^2^ over 0.99. All shown error bars represent standard deviations (*n* = 4).

Biophotoelectrodes were constructed by adsorbing purified RCs onto bare nanostructured silver (Ag^R^) electrodes, prepared as previously described.^24^ The surface architecture provided an ample surface area for increasing the loading of RCs and cyt *c*,^24^ which has been shown as an effective strategy to boost photocurrents.^28^ The proposed arrangement of proteins on the electrode, as well as the mechanism of the electron transfer pathway, is depicted in Figure 2. The biophotoelectrode activity was measured in an electrochemical cell containing 1.5 mM water-soluble ubiquinone-0 (Q_0_) as an electron acceptor at an applied potential of +160 mV versus the standard hydrogen electrode (SHE). To exclude acceptor side (i.e., Q_B_/Q_0_) limitations or short-circuits from controlling RC turnover,^4^ the light intensity was lowered to 2.6 mW cm^–2^ such that peak photocurrents were in a linear regime with respect to the light intensity (Figure S2). To characterize dependence on its concentration, cyt *c* was titrated into the electrolyte, and, after equilibration, the photocurrent during 30 s of illumination was recorded (Figure 3a). The size of the photocurrent increased with the concentration of added cyt *c* until a plateau was reached above 20 μM (Figure 3b and Table 1). This titration was repeated for electrodes coated with each of the three engineered RCs (Figure S3). For all three, an increase in photocurrent was seen as the concentration of cyt *c* was increased (Figure 3b), but overall, they produced smaller photocurrent densities than those seen for the WT RC (Figure 3b and Table 1). In an electrolyte containing 20 μM cyt *c*, the photocurrent was found to be highly stable, decreasing negligibly over four consecutive photocurrent recordings (Figure S4).

As photocurrent density will be dependent on the quantity of RC that was adsorbed to each electrode, pigments were extracted from the electrode and quantified by absorbance spectroscopy (see Methods). RC loadings (Γ_RC_) varied between approximately 50 and 80 pmol cm^–2^ (Table 1), likely stemming from differences between preparations of concentrated RCs, such as the final detergent concentration, or minor variations in the electrode preparation process. These loadings were used to calculate values of the RC turnover frequency (TOF) for the cyt *c* titrations (Figure 3c). The maximal RC TOFs for WT RCs and the Asp-M88Lys RC with weakened cyt *c* binding were comparable (Table 1), whereas the two RCs with strengthened cyt *c* binding achieved significantly lower TOFs. This suggested that increasing the affinity of the RC for cyt *c* was detrimental for electron transfer and photocurrent generation. The Hill equation was used (see Methods) to obtain the cyt *c* concentration that corresponded to the half-maximal photocurrent (*K*_PC_). This *K*_PC_ fell in a small range between 2.3 and 3.6 μM cyt *c* for all RC variants including the WT protein, in stark contrast to the *K*_D_ values that spanned nearly four orders of magnitude (Table 1).

**Figure 4:**
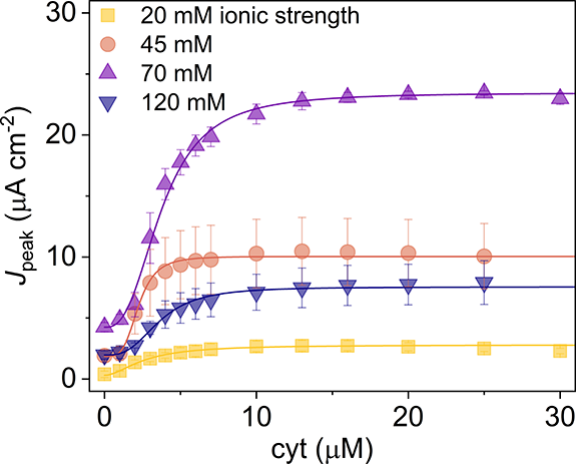
Peak photocurrents at different ionic strengths as a function of cyt *c* concentration. In addition to KCl, the electrolyte buffer also contained 20 mM Tris-Cl, which was included in the ionic strength calculation. Lines show Hill equation fits of the data, all of which converged with an *R*^2^ > 0.99.

The effect of electrolyte ionic strength on photocurrent output by WT RCs was also examined, again as a function of cyt *c* concentration (Figure 4). Ionic strength has a number of potential influences, including promoting mobility of cyt *c* at the electrode surface and screening of electrostatic interactions between the cyt *c* and the RC that are important for docking. Variation of ionic strength had a marked effect on the maximal photocurrent output, with a local optimum at 70 mM KCl (Figure 4). Photocurrents decreased at 120 mM ionic strength, likely due to cyt *c* desorption as reported previously on a SAM-functionalized electrode.^21^

**Figure 5:**
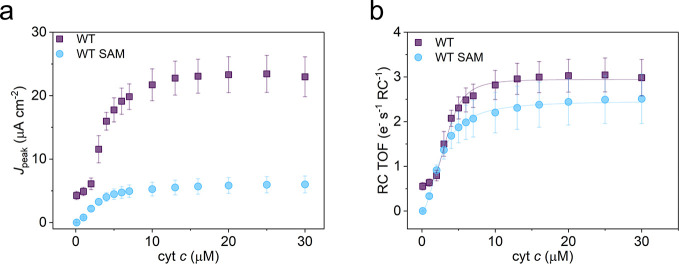
Electrode functionalization. (a) Peak photocurrents are shown as a function of solution cyt *c* concentration from WT RCs adsorbed onto a bare or SAM-functionalized Ag^R^ electrode. The SAM consisted of a 3:1 ratio of mercaptoundecanol and mercaptoundecanoic acid. (b) RC TOF as a function of cyt *c* concentration. Error bars represent standard deviations (*n* = 3). Lines show Hill equation fits, all of which converged with an *R*^2^ over 0.99.

To probe whether electrode–cyt *c* interactions affect RC turnover, prior to RC deposition, electrodes were coated with a self-assembled monolayer (SAM) comprising mercaptoundecanoic acid and mercaptoundecanol in a 3:1 ratio.^21^ This SAM is terminated by carboxylic acid and hydroxyl residues that result in a negatively charged surface that should promote oriented cyt *c* adsorption, with the heme facing toward the electrode surface.^29^ The SAM would be expected to modulate the electrode–cyt *c* interface but not the RC/cyt *c* interface, isolating changes in the RC TOF to changes in electrode–cyt *c* interaction. In titrations with cyt *c*, photocurrents from the SAM-functionalized electrodes plateaued at ∼6 μA cm^–2^, four-fold lower than the ∼24 μA cm^–2^ achieved in the absence of a SAM (Figure 5a). However, much of this decline could be accounted for by lower RC loadings (Γ_RC_), such that the difference in the RC TOF between the two surfaces was not statistically significant (Figure 5b and Table S1). Interestingly, the cyt *c* titration curve on the SAM-coated electrode revealed a *K*_PC_ of 2.8 μM cm^–2^, similar to the 3.6 μM cm^–2^ achieved on a bare Ag^R^ electrode, suggesting that the two surfaces were similar in their affinity for cyt *c*.

## Discussion

Although purple bacterial RCs and larger RC–LH1 complexes can produce photocurrents when directly interfaced with an electrode, it is well-established that the use of cyt *c* as a mediator can boost photocurrents.^5,19,24^ In contrast to the role played by quinones in mediating electron flow from the “negative terminal” of the RC to a counter electrode, which has been studied in detail,^30^ the mechanism by which cyt *c* achieves mediation to the “positive terminal” of the RC remains poorly understood. In nature, cyt *c*_2_ enables RC reduction during repetitive charge separation events by, following electron donation to P870^+^, detaching and diffusing through the periplasmic space to be rereduced by the intramembrane cyt *bc*_1_ complex. The overall process is therefore dependent on two specific and transient protein–protein interactions, one at the RC/cyt *c* interface and one at the cyt *bc*_1_/cyt *c* interface, as well as diffusion between the two. On an electrode, the details of the equivalent interactions are less well-understood, other than knowing that cyt *c* must make sufficiently intimate contacts with both the RC and the underlying electrode to mediate electron transfer between the two.

**Figure 6:**
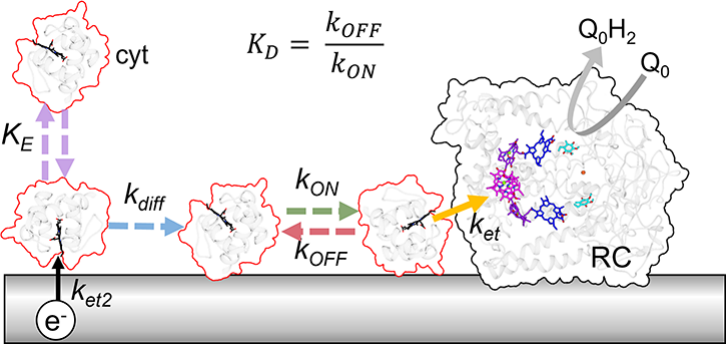
Electrostatic interfaces and the proposed mechanism of electron transfer. This schematic depicts the adsorption and desorption of cyt *c* (mauve dashed arrows) onto an electrode until a binding equilibrated concentration (*K*_E_) is reached. The cyt *c* heme is depicted in black. Following electrode reduction of oxidized cyt (*k*_et2_/black solid arrow), cyt *c* diffuses (blue dashed arrow) and binds to the RC (green arrow) at a rate *k*_ON_. An electron is transferred from cyt *c* to the photo-oxidized P870^+^ (yellow solid arrow) at a rate *k*_et_. Finally, oxidized cyt *c* dissociates from the RC (red dashed arrow) at a rate *k*_OFF_. Dashed arrows indicate diffusional processes, and solid arrows represent electron transfers.

As a minimum (Figure 6), interfacing of RCs and cyt *c* with electrodes for effective solar energy conversion requires balancing of the interaction between cyt *c* and the electrode (*K*_E_) with the interaction between cyt *c* and the RC (*K*_D_). The two electrostatic interfaces control the kinetics of electron transfer and RC turnover, which in turn dictate the magnitude of the photocurrent and the efficiency of solar energy conversion. As depicted in Figure 6, it is also possible that mobility of cyt *c* on the electrode surface (*k*_diff_) has an influence, recapitulating the situation in natural photosynthesis. A number of aspects of this system remain unclear, including the extent to which long-range mobility of cyt *c* is important, whether cyt *c* needs to orient in a specific fashion to collect an electron from the electrode and then reorient to deliver it to the photo-oxidized RC, and the extent to which the RC/cyt *c* interface operates in a manner analogous to that well-characterized in the natural system.

The primary aim of this study was to examine whether single residue alterations in the RC interaction surface known to change the strength of cyt *c* binding would have any effect on the photocurrent sustained by the RC. For example, it could be postulated that, if mobility of cyt *c* is not important (i.e., the system is “hard-wired”), strengthening binding could increase a photocurrent while weakening binding could decrease it. Alternatively, if cyt *c* mobility is important (Figure S5a), then strengthening binding by the RC might decrease the current. The lack of any effect of the mutations could indicate that the RC–cyt *c* interaction is different from that characterized in the natural system.

The data obtained with the Asn-M188Asp and Gln-L264Glu RCs, both of which strengthen binding of cyt *c* by making the RC interaction surface more electronegative, would seem to rule out the last of these proposals. Both lowered the maximum RC TOF identifiable in cyt *c* titrations (Table 1), indicating that single residue changes in the cyt *c* binding site have a discernable impact and enabling the conclusion that cyt *c* has to interact with this part of the RC protein to deliver electrons from the electrode. The fact that strengthening binding did not increase the photocurrent also argues against a model where an immobile cyt *c* hardwires electron transfer (Figure S5b). The data are most consistent with a picture in which mobility within the cyt *c* layer is important for photocurrent generation (Figure S5a).

As can be seen in Table 1, the lower cyt *c* binding affinity RC mutant Asp-M88Lys (*K*_D_ = 55 μM compared to 0.3 μM for the WT RC) achieved a maximum RC TOF that was only marginally lower than that achieved by the WT RC. While this mutant exhibited a slower docking rate (*k*_ON_) *in vitro*,^18^ photocurrents were not significantly different from that from WT RCs, suggesting that a lower cyt *c* docking rate was not a rate-limiting step in the biophotoelectrode. Conversely, the higher cyt *c* binding affinity mutants Asn-M188Asp (*K*_D_ = 0.06 μM) and Gln-L264Glu (*K*_D_ = 0.01 μM) both exhibited significant decreases in the maximum RC TOF. Since these mutants displayed a substantially slower *k*_OFF_ vs WT *in vitro*, these data suggest that undocking of cyt *c* from the RC may be a rate-limiting step. It is noteworthy that the *k*_OFF_ rates reported for these mutants in solution were much faster than the observed electrode RC TOF, leading one to conclude that *k*_OFF_ should not be rate-limiting on an electrode. However, the additional interaction of both the RC and the cyt *c* with the electrode may decrease docking and undocking rates relative to *k*_OFF_ values reported *in vitro* and explain our observations.

Cyt *c* was titrated into the electrolyte to test a hypothesis that the formation of a stable cyt–RC complex on an electrode would drive RC turnover (Figure S5b). In this configuration, electrons would tunnel from the electrode to the cyt *c* heme for subsequent transfer to the RC P870^+^ without cyt *c* undocking from the RC (Figure S5b).^5,31^ According to this model, the formation of the RC–cyt *c* complex should be directly proportional to the photocurrent, and the half-maximal cyt *c* concentration (*K*_PC_) would mirror *K*_D_. In all RC mutants, we found that a cyt *c* concentration of ∼3 μM cyt *c* resulted in a half-maximal photocurrent output (*K*_PC_). The observed values of *K*_PC_ were in marked contrast to the expected RC–cyt *c* binding affinities (*K*_D_) measured in previous solution-based experiments^6^ (Table 1, *K*_PC_ vs *K*_D_). This indicates that the dependence of photocurrent on cyt *c* concentration is not correlated with RC–cyt *c* binding, but rather an independent event, which we attributed to the binding of cyt *c* to the electrode (*K*_E_ in Figure 6).^21^ The result ultimately supports the view that RCs cannot be “wired” to the electrode via cyt *c* in a static configuration (Figure S5b)^5^ but are primarily dependent upon the loading of the electrode with mobile cytochromes. According to this model, the RC would be attached to the electrode directly, and cyt *c* would relay electrons from the electrode to the RC (Figure S5a). This finding is in agreement with previous results using WT RCs on a SAM-coated electrode, whereby cross-linking and immobilization of cyt *c* halted RC turnover.^21^

The electrostatic binding interface was probed by functionalizing the silver electrode with a self-assembled monolayer (SAM) that favorably binds and orients the cyt *c* such that the heme cleft faces the electrode.^32^ The absolute photocurrent was much smaller on the SAM-functionalized electrode, but this was caused by a decrease in RC loading, suggesting that the binding affinity of the SAM-functionalized electrode for the RC was diminished. Nevertheless, alteration of this electrode–cyt *c* interface did not produce significant changes in the RC TOF, suggesting that surface functionalization had little effect on the kinetics of cyt *c* electron transfer and mobility relative to a bare electrode.

Given that *K*_PC_ for the SAM-coated electrode was similar to the *K*_PC_ for the unfunctionalized electrode, we suggest that the two surfaces have very similar cyt-binding affinities and, hence, similar electrostatic interactions. Since cyt *c* does not give a clear CV on bare metal, we could not quantify the cyt *c* loading on the bare electrode to directly confirm that *K*_E_ is equal to *K*_PC_. However, previous findings on SAM-coated electrodes reveal that cyt *c* coverage on the electrode is directly proportional to the photocurrent, whereby *K*_E_ is equal to *K*_PC_.^21^ Overall, the result suggests that cyt *c* electrode adsorption is the major determinant that explains the shape of the photocurrent titration curves and that an electrode maximally saturated with cyt *c* is beneficial for photocurrent output in the current biophotoelectrode configuration.

Cyt *c* mobility (*k*_diff_) on the electrode may also play a significant role in restricting RC turnover (Figure 6). Increasing the ionic strength of the electrolyte buffer would promote cyt *c* mobility by screening the electrostatic binding interactions between cyt *c* and the electrode. However, higher ionic buffer strengths also cause desorption of cyt *c* from the electrode, effectively lowering the cyt *c* concentration at the electrode-confined RCs (Figure 4). Furthermore, higher ionic strengths screen the interactions between the RC and cyt, preventing efficient cyt–RC docking.^20^ At low ionic strengths of 20 and 45 mM, we found that photocurrents were small but increased to 23 μA cm^–2^ at 70 mM, clearly demonstrating the beneficial effects of electrostatic screening to boost photocurrents. However, at a higher concentration, the photocurrent drops off again, likely due to the desorption of cyt, as demonstrated previously on a SAM electrode.^21^ We hypothesize that this increased photocurrent at 70 mM ionic strength stems from an increased mobility of cyt *c* on the electrode and not from an increased undocking rate of cyt *c* from the RC since the mutant Asn-M88Lys with a more rapid *k*_OFF_ did not result in an increased RC TOF relative to WT. A further increase of the ionic strength resulted in a decrease in photocurrents, which we attribute to either desorption of the cyt *c* from the electrode, in agreement with previous results on a SAM-coated electrode.^21^ We can exclude a reduction in the cyt–RC association rate (*k*_ON_) at higher ionic strengths, as these rates are still very high in comparison with *k*_OFF_ and the RC TOF observed on the electrode.

Mediators that are not desorbed from the electrode at high ionic strengths, such as cross-linked osmium redox polymers, have recently been identified as effective matrices to drive efficient forward electron transfer to RCs, with solar-to-chemical conversion efficiencies of ∼50%,^33^ and photosystem I turnover frequencies of over 300 e^–^ s^–1^.^1,34^ However, such redox polymers require potentially toxic heavy metals such as osmium, the least abundant element in Earth’s crust. A scalable and sustainable mediator such as cyt *c* could be of interest in biohybrid applications if the efficiencies of electron relay could be brought on par with those of high-performing osmium redox polymers.

## Conclusions

This work investigates how the electrostatic interfaces influence RC turnover in a biohybrid photoelectrode. Amino acid substitutions at the RC binding interface that promoted stronger cyt *c* binding resulted in significant decreases in RC turnover, suggesting that the rate of dissociation of cyt *c* from the RC became rate-limiting. Conversely, turnover of a mutant RC with lower cyt *c* binding affinity was not significantly different from the WT RC, suggesting that the docking rate was not limiting. Photocurrents were mainly dependent on the cyt–electrode loading and not on cyt-RC binding, suggesting that direct wiring of RCs directly to electrodes via cyt *c* is not feasible and that a large pool of mobile cyt *c* is beneficial for RC turnover. Lastly, a strong influence of ionic strength on photocurrent output was found, which suggests that increasing cyt *c* mobility is beneficial for RC turnover. The photocurrent decreased again at 120 mM ionic strength, likely due to desorption of cyt *c* from the electrode. The data suggest that mobility of electrode-adsorbed cytochromes, which dock and undock from the RC, is supportive of RC turnover and that future biohybrid electrodes may be improved by targeting cyt *c* mobility while preventing cyt *c* desorption from the electrode.

## Materials and Methods

### Materials

Horse heart cyt *c*, 2,3-dimethoxy-5-methyl-*p*-benzoquinone (Q_0_), mercaptoundecanoic acid, and mercaptoundecanol were purchased from Sigma-Aldrich. Milli-Q water (Millipore, MA) was used in all preparations and procedures. Planar disc 2 mm Ag electrodes were purchased from CH Instruments, Austin, TX. Reference electrodes, counter electrodes, and potentiostats were purchased from Metrohm Autolab BV, Utrecht, Netherlands. A high-power multiarray LED (870-66-60) centered at 870 nm was purchased from Roither-Lasertechnik GmbH, Wien, Austria.

### RC Isolation and Purification

His-tagged WT RCs were purified by nickel affinity chromatography and size exclusion chromatography from a strain of *Rba. sphaeroides* lacking light-harvesting complexes, as described previously.^2^ RCs with site-directed mutations Asp-M88 to Lys, Asn-M188 to Asp, or Gln-L264 to Glu were constructed as previously described and purified in the same way.^2,18^

### Electrode Construction

Nanostructured silver (Ag^R^) electrodes were fabricated as previously described.^24^ Briefly, planar disc 2 mm Ag working electrodes (Metrohm) were mechanically polished with Al_2_O_3_ lapping films of successively finer grain sizes of 5, 3, and 1 μm (Thorlabs) followed by rinsing of the electrode with Milli-Q water after each polishing step. An electrochemical roughening procedure was then applied to create Ag^R^ electrodes, as described previously.^24,35^ Electrodes coated with a SAM of mercaptoundecanoic acid and mercaptoundecanol were prepared as previously described.^21^

### RC Adsorption

The four RC variants were solubilized in 20 mM Tris, pH 8.0, 0.04% w/v dodecyl-beta-d-maltoside and diluted to a concentration of 46.3 μM. The Ag^R^ electrodes were incubated in these RC solutions for 1 h in the dark at 4 °C. The electrodes were then incubated in 1 M KCl and 5 mM Tris buffer (pH 8.0) for 10 min followed by incubation in 20 mM Tris buffer (pH 8.0) for another 10 min to remove any trace cyt *c* from the RC preparation. Both incubations took place in the dark at room temperature. All experiments had a sample size *n* = 3 or more as indicated. Addition of cyt *c* followed RC adsorption. In contrast to previous spectroscopic studies *in vitro*, the native *Rba. sphaeroides* cyt *c*_2_ was substituted by the commercially available equine horse heart cytochrome *c*. Mammalian cyt *c* has been demonstrated as a functional substitute for the bacterial cyt *c*_2_ both *in vitro* and in wiring RCs to electrodes.^5,13^ Horse heart cyt *c* exhibits a *K*_D_ of 0.4 μM with RCs *in vitro*, which is similar to 0.3 μM for the native bacterial cyt *c*_2_, enabling comparison between studies that utilize bacterial cyt *c*_2_*in vitro* and mammalian cyt *c* on an electrode.^20^

### Photocurrents

The loaded Ag^R^ electrodes were inserted into a photoelectrochemical cell fitted with a Ag/AgCl reference electrode and a platinum counter electrode (Autolab Metrohm). A PGSTAT128N potentiostat (Metrohm) was used to control the three-electrode cell, with a bias potential of −50 mV vs Ag/AgCl being applied. The three-electrode cell was filled with an electrolyte containing 20 mM Tris buffer (pH 8.0), 50 mM KCl, and 1.5 mM Q_0_, and the concentration of cyt *c* was indicated. Illumination was provided by an LED centered at 870 nm at an intensity of 2.9 mW cm^–2^. A shutter in between the LED and the three-electrode cell determined whether the cell was illuminated.

### Cyt *c* Titrations

After the RC adsorption on the Ag^R^ electrodes, the peak photocurrent of each electrode was measured at an increasing concentration of cyt *c* in an electrolyte containing 20 mM Tris buffer (pH 8.0), 50 mM KCl, and 1.5 mM Q_0_. Electrodes were inserted into a photoelectrochemical cell and allowed to equilibrate for 100 s. After this equilibration period, a chronoamperogram was measured for 80 s, during which the shutter in between the LED and the three-electrode cell was opened for 30 s. After this complete 180 s period, the electrolyte was removed from the cell, and an aliquot of cyt *c* was added to the electrolyte and thoroughly mixed to establish a predetermined cyt *c* concentration. The electrolyte was then again back to the cell, after which the mentioned 180 s period started again. This process was repeated to measure the photocurrents at an increasing range of cyt *c* concentrations. All photocurrent measurements were performed under ambient conditions, in air, and at room temperature. Thorough mixing after cyt *c* titration and a three-minute incubation period were added to ensure that equilibrium was reached between free and electrode-bound cyt. This was verified by observing that any additional incubation time did not result in significant increases in the photocurrent response. Cytochrome *c* was dissolved in an electrolyte containing 20 mM Tris buffer (pH 8.0), 50 mM KCl, and 1.5 mM Q_0_ using vigorous vortexing and 2 min of sonication on ice. The stock was made fresh on the day of the experiment.

### Fitting with the Hill Equation

Data from measurements were fitted with the Hill equation, which adds an extra term to the Michaelis–Menten equation to account for the positive cooperativity (*n*) that has been observed in cyt *c* biophotoelectrochemical systems.^21^ This cooperativity has been previously shown to stem from cyt *c* more effectively funneling electrons to the electrode-adsorbed RCs with an increasing cyt *c* electrode loading.^21^

### Determination of the RC Loading

Electrodes were inserted into a microcentrifuge tube containing 200 μL of 80% acetone/20% water and vortexed for 30 s in the dark followed by mild sonication for 30 s. The electrode was removed, the sample was centrifuged at 10,000 RCF for 5 min, and the absorbance spectrum of the solution containing extracted bacteriochlorophyll was recorded with a PerkinElmer Lambda 40 spectrometer. The loading of RC complexes on the electrode (Γ_RC_, mol cm^–2^) was calculated using an extinction coefficient of 69 mM^–1^ cm^–1^ at 770 nm, assuming four bacteriochlorophyll pigments per RC.^9^ The contribution of bacteriopheophytin was deconvoluted and subtracted from the pigment extraction spectrum.

### TOF Calculation

The RC TOF was calculated as previously described,^21^ using the following equation:
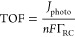
1where *J*_photo_ is the photocurrent density in A cm^–2^, Γ_RC_ is the RC loading in mol cm^–2^, *F* is the Faraday constant (96,485 C mol^–1^), and *n* is the number of electrons per cyt *c* turnover. The apparent RC turnover rates assumed that the activity of wired RCs was 100%.

### Hill Fit of TOF–Cyt *c* Titration Curves

Data were fitted in OriginLab using the Hill equation:
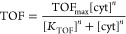
2where TOF_max_ is the maximal RC turnover, *K*_PC_ is the half-maximal cyt *c* concentration constant, and *n* is the Hill coefficient.

## Supporting Information Available

Mechanism of RC–cyt electron transfer *in vitro*, biphasic P870^+^ reduction kinetics, illumination intensity and photocurrents, photocurrent transients and stability, and proposed mechanisms of cyt-mediated electron transfer (PDF)
