# Supplementary material for: The Role of Electrostatic Binding Interfaces in the Performance of Bacterial Reaction Center Biophotoelectrodes
Source: ACS Sustain Chem Eng. 2023 Feb 7;11(7):3044–51. doi: 10.1021/acssuschemeng.2c06769 (PMC9945312; doi:10.1021/acssuschemeng.2c06769)
Supplement: Supplementary file 1 — sc2c06769_si_001.pdf [file sc2c06769_si_001.pdf]

Supporting Information

The role of electrostatic binding interfaces in the performance of bacterial reaction center biophotoelectrodes

Milo R. van Moort[1,2], Michael R. Jones[3], Raoul N. Frese[1,2], Vincent M. Friebe[1,2,4]*

ORCiDs, emails:
MRM 0000-0001-7015-0475 milovanmoort@hotmail.com
RNF 0000-0001-8243-9954 r.n.frese@vu.nl
MRJ 0000-0002-8063-0744 m.r.jones@bristol.ac.uk
VMF 0000-0002-4277-8473 vincent.friebe@tum.de

Affiliations
[1] Biophysics of Photosynthesis, Department of Physics and Astronomy, Faculty of Science, Vrije Universiteit Amsterdam, 1081 HV Amsterdam, The Netherlands
[2] LaserLaB Amsterdam, Vrije Universiteit Amsterdam, 1081 HV Amsterdam, The Netherlands
[3] School of Biochemistry, Biomedical Sciences Building, University of Bristol, University Walk, Bristol BS8 1TD, United Kingdom
[4] Campus Straubing for Biotechnology and Sustainability, Technical University of Munich, Uferstraße 53, 94315 Straubing, Germany

* To whom correspondence should be addressed vincent.friebe@tum.de

Number of pages: 9

Number of Tables: 1

Number of Figures: 5

Mechanism of RC-cyt electron transfer *in vitro*. A simplified scheme detailing light-induced charge separation and electron transfer is shown in Figure S1. Prior to illumination, reduced cyt *c* (cyt^{red}) and RCs are in an equilibrium between free and bound states. The binding constant (K_B) is given by:

$$K_B = \frac{1}{K_D} = \frac{[\text{RC:cyt}^{red}]}{[\text{cyt}^{red}][\text{RC}]} \qquad (S1)$$

whereby [cyt^{red}] and [RC] are the concentrations of the free proteins, and [RC:cyt^{red}] is the concentration of bound complexes. The equilibrium state is determined by the strength of the binding interaction between the proteins, which comprises electrostatic attraction and complementary hydrophobic patches.[1–3] The strength of an electrostatic attraction can be mitigated by the ionic strength of the electrolyte through screening of charges.[4] The dissociation constant (K_D) is the inverse of the association constant, which is often used to define binding affinity. [2,5,6] It is related to the rate of binding (k_{ON}) and unbinding (k_{OFF}) according to:

$$K_D = \frac{k_{OFF}}{k_{ON}} \qquad (S2)$$

Parameters k_{ON} and k_{OFF} have units of $M^{-1} \cdot s^{-1}$ and s^{-1}, respectively. A K_D of ~0.3 µM has been experimentally determined for wild-type RCs and cyt c_2.[2]

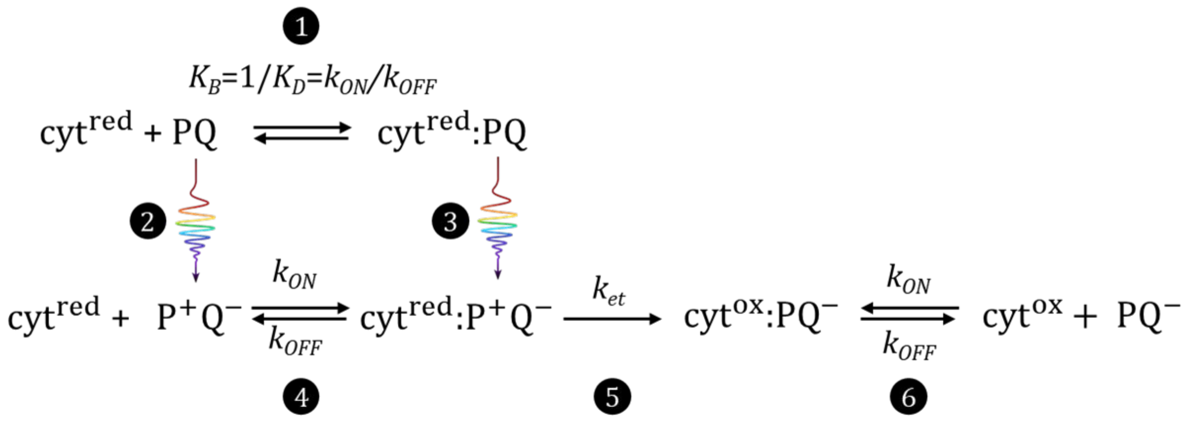

Figure S1. Mechanism and kinetics of RC-cyt *c* electron transfer *in vitro*. A schematic detailing a single charge separation and electron transfer event, adapted from.[2] P870 is abbreviated to P. Reversible association of reduced cyt *c* (cyt^{red}) with the RC can take place either before (1) or after (4) photoexcitation (2,3). This binding interaction is described by a binding constant (K_B), dissociation constant (K_D), and rate constants for binding (k_{ON}) and unbinding (k_{OFF}). Photoexcitation (2,3) results in charge separation and formation of the oxidized bacteriochlorophyll pair and reduced quinone acceptor (P^+Q^-). This is followed by reduction of P^+ by cyt^{red} (5) at the rate k_{et}. The unbinding of oxidized cyt *c* (cyt^{ox}) enables cytochrome exchange.

Biphasic P870$^+$ reduction kinetics. Experimentally it has been found that the kinetics of P870$^+$ reduction by cyt^{red} is biphasic, due to the possibility that P870 can be photooxidized regardless of whether the RC has a bound cyt^{red} or not. In the fraction of RCs that are bound to cyt^{red} when photoexcitation occurs, P870$^+$ is reduced by the bound cyt^{red} with a lifetime of around one microsecond, and so is described by a first-order rate constant ($k_{et} \approx 10^6$ s^{-1}).[5,7] The remaining fraction of free RCs must first dock with a free cyt^{red} before P870$^+$ reduction can occur, resulting in a slower kinetic component that is dependent on the concentration of cyt *c* . This component is described by a second-order electron transfer rate constant (k_2) according to:

$$k_2 = \frac{k_{et} k_{ON}}{k_{OFF} + k_{et}} \qquad \text{(S3)}$$

where k_{et} is the first-order electron transfer rate within the bound complex. The experimentally determined second-order rate constant k_2 approximates to k_{ON} at low ionic strength (10 mM), since $k_{et} >> k_{OFF}$ under these conditions.[1,5] Determination of k_{ON} in this way enables calculation of k_{OFF} using equation S2, which is useful in identifying potentially rate-limiting steps (Table 1). The rate of the second-order reaction also decreases at increasing ionic strengths[8], consistent with the electrostatic screening of the long-range RC-cyt *c* binding interface under high ionic strengths, and forms a crucial parameter to consider when comparing rate constants.[4,9]

Illumination intensity and photocurrents. Losses in biophotoelectrode systems are primarily due to slow interfacial electron transfer and diffusion-limited mass transport, the latter of which leads to depletion of the electron acceptor and charge recombination of its reduced form with the electrode.[10,11] As the focus of the present work was probing cyt-mediated electron transfer between the RC and the electrode, the intensity of the actinic light was lowered and a relatively high concentration of Q_0 was used such that acceptor side limitations were not limiting, such that donor side limitations could be isolated. As illustrated in Figure S2, photocurrents exhibit a relatively linear relationship with irradiance in a low light regime.[12] Accordingly, a light intensity of 2.9 mW cm^{-2} was used to measure photocurrents. Despite linearity in peak photocurrent values, the stable photocurrents were still noticeably lower than peak photocurrents (Figure S3), likely extending from charge recombination of the accumulate photo reduced product (hydroquinone) with the electrode.

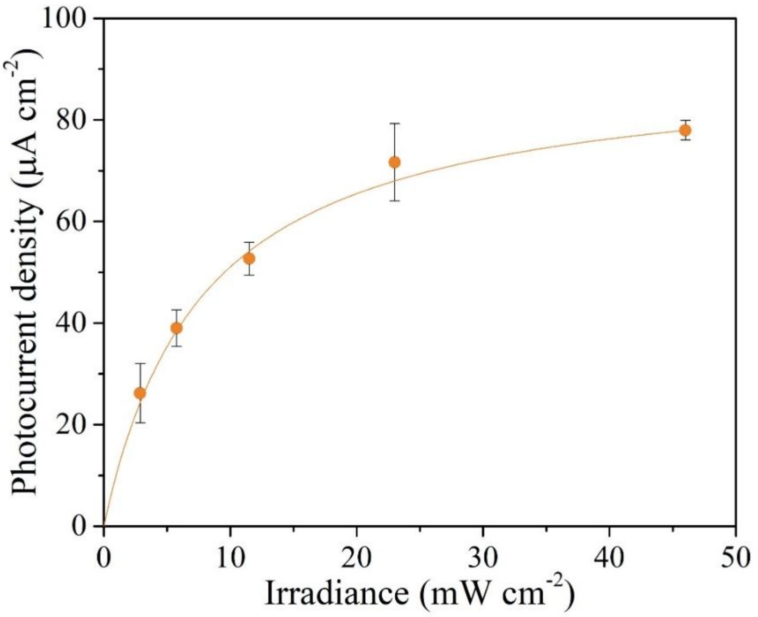

Figure S2. Photocurrents and irradiance. Peak photocurrents for WT RCs on Ag^R electrodes at different irradiance intensities. The data are fitted with the Michaelis Menten equation with an R^2 > 0.99. Errors are indicated by bars, n=3

Photocurrent transients. Photocurrent transients recorded during cyt c titrations are shown in Figure S3. The data for WT RCs are also shown in Figure 3a. Photocurrents were typically maximal shortly after illumination started and then declined due to mass transport limitations associated with the Q_0/Q_0H_2 mediator [10,12,13]. The spike of negative current seen after the cessation of illumination is attributed to recombination of Q_0H_2 with the working electrode[13].

For WT RCs, it was notable that small photocurrents with a peak value of approximately 5 μA cm^{-2} were observed in the absence of free cyt on bare rough silver, but not on the SAM functionalized electrode (Figure S3). We hypothesize that the small photocurrent on bare Ag^R in the absence of free cyt stems from either a small population of RCs directly wired to the electrode, or electron transfer between the two mediated by quinone/quinol. Since the SAM likely blocks both direct RC-electrode ET, as well as blocking quinone-mediated ET as evidenced by the absence of a reverse photocurrent spike in the presence of the SAM (Figure S3), it is not possible to exclude either of these possible ET pathways.

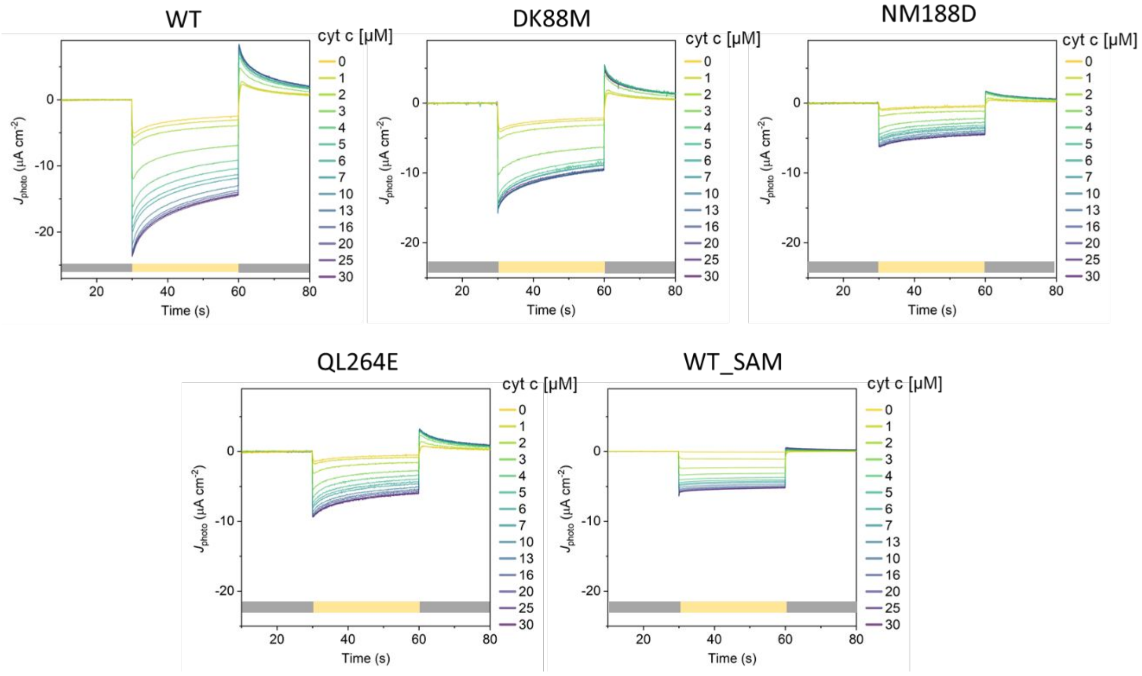

Figure S3. Photocurrent transients for WT and mutant RCs. WT-SAM denotes WT RCs on a SAM-coated silver electrode. Periods of dark and illumination are indicated by the gray and yellow bars. The concentrations of cyt *c* for each photocurrent trace are shown in the legend.

Photocurrent Stability. The stability of the photocurrent was monitored and found to decrease negligibly over the course of four photocurrent recordings, as depicted below in Figure S4.

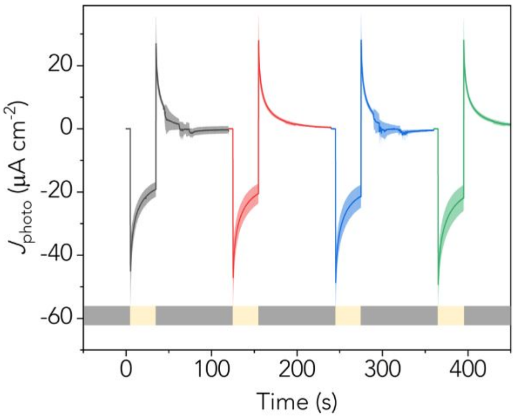

Figure S4. **Photocurrent transients for WT RCs**. Irradiance equaled 12.5 mW cm^{-2} (870 nm), and photocurrents were measured at -50mV vs AgAgCl in an electrolyte containing 20 mM Tris / pH 8.0 / 50 mM KCl and 20µM cyt *c*. Periods of dark and illumination are indicated by the gray and yellow bars, respectively.

Table S1. Experimental data for WT RCs on bare and functionalized silver electrodes.

	K_{PC} (µM)	J_{photo} (µA cm^{-2})	Γ_{RC} (pmol cm^{-2})	TOF (e$^-$ s^{-1} RC^{-1})
WT on bare Ag^R	3.6 ± 0.1	23 ± 4.0	80 ± 3	3 ± 0.3
WT on SAM-Ag^R	2.8 ± 0.1	5.8 ± 1.2	25 ± 2	2.7 ± 0.5

Data shown are the RC-cyt dissociation constant in BioPEC systems (K_{PC}), peak photocurrent (J_{photo}), RC-loading (Γ_{RC}) and electron turnover frequency (TOF) for the WT and the WT on a SAM. All values are shown with their standard deviation (n=3).

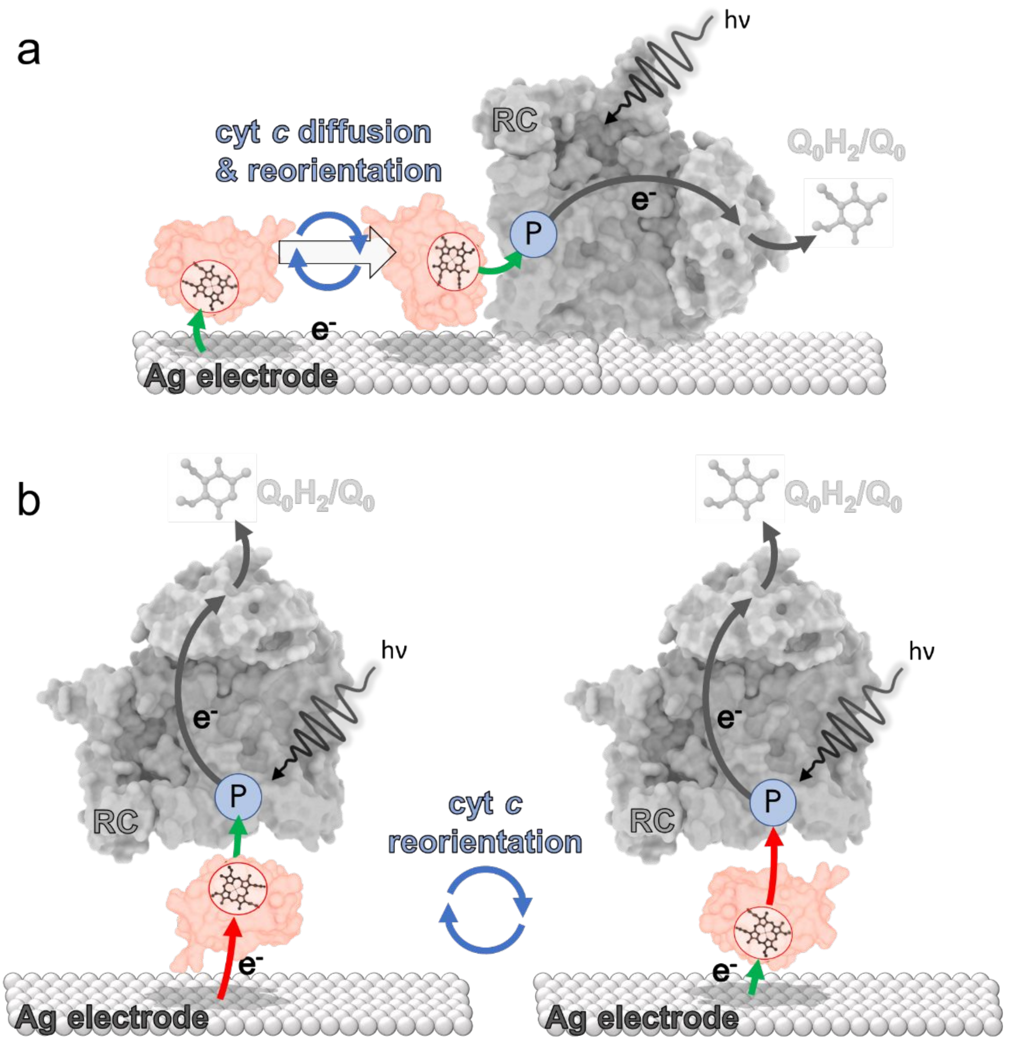

Figure S5. Proposed mechanisms of cyt-mediated electron transfer. a) a mechanism where cyt must orient the heme in an optimal position to facilitate electron transfer from the electrode substrate, diffuse across the electrode surface, and dock to the RC with a new optimal orientation to transfer an electron. Facile electron transfer steps indicated in green arrows. b) An alternative proposed mechanism whereby the RC is 'wired' to the electrode via docking to a cyt. Fast electron transfer (green arrows) requires a short electron tunneling distance. Reorientation could be required to prevent slow, long-distance electron transfer (red arrows) becoming limiting.

References:

(1) Abresch, E. C.; Gong, X. M.; Paddock, M. L.; Okamura, M. Y. Electron Transfer from Cytochrome c_2 to the Reaction Center: A Transition State Model for Ionic Strength Effects Due to Neutral Mutations. *Biochemistry* **2009**, *48* (48), 11390–11398. DOI: 10.1021/bi901332t.

(2) Tetreault, M.; Rongey, S. H.; Feher, G.; Okamura, M. Y. Interaction between Cytochrome c_2 and the Photosynthetic Reaction Center from Rhodobacter Sphaeroides: Effects of Charge-Modifying Mutations on Binding and Electron Transfer. *Biochemistry* **2001**, *40* (29), 8452–8462. DOI: 10.1021/bi010222p.

(3) Tetreault, M.; Cusanovich, M.; Meyer, T.; Axelrod, H.; Okamura, M. Y. Double Mutant Studies Identify Electrostatic Interactions That Are Important for Docking Cytochrome c_2 onto the Bacterial Reaction Center. *Biochemistry* **2002**, *41* (18), 5807–5815. DOI: 10.1021/bi012053e.

(4) Wachtveitl, J.; Farchaus, J. W.; Mathis, P.; Oesterhelt, D. Tyrosine 162 of the Photosynthetic Reaction Center L-Subunit Plays a Critical Role in the Cytochrome c_2 Mediated Rereduction of the Photooxidized Bacteriochlorophyll Dimer in Rhodobacter Sphaeroides. 2. Quantitative Kinetic Analysis. *Biochemistry* **1993**, *32* (40), 10894–10904. DOI: 10.1021/bi00091a045.

(5) Tiede, D. M.; Vashishta, A. C.; Gunner, M. R. Electron-Transfer Kinetics and Electrostatic Properties of the Rhodobacter Sphaeroides Reaction Center and Soluble *c*-Cytochromes. *Biochemistry* **1993**, *32* (17), 4515–4531. DOI: 10.1021/bi00068a006.

(6) Jarmoskaite, I.; Alsadhan, I.; Vaidyanathan, P. P.; Herschlag, D. How to Measure and Evaluate Binding Affinities. *Elife* **2020**, *9*, 1–34. DOI: 10.7554/ELIFE.57264.

(7) Axelrod, H. L.; Abresch, E. C.; Okamura, M. Y.; Yeh, A. P.; Rees, D. C.; Feher, G. X-Ray Structure Determination of the Cytochrome c_2: Reaction Center Electron Transfer Complex from Rhodobacter Sphaeroides. *J. Mol. Biol.* **2002**, *319* (2), 501–515. DOI: 10.1016/S0022-2836(02)00168-7.

(8) Gerencsér, L.; Laczkó, G.; Maróti, P. Unbinding of Oxidized Cytochrome c from Photosynthetic Reaction Center of Rhodobacter Sphaeroides Is the Bottleneck of Fast Turnover. *Biochemistry* **1999**, *38* (51), 16866–16875. DOI: 10.1021/bi991563u.

(9) Prince, R. C.; Cogdell, R. J.; Crofts, A. R. The Photo-Oxidation of Horse Heart Cytochrome c and Native Cytochrome c_2 by Reaction Centres from Rhodopseudomonas Spheroides R26. *Biochim. Biophys. Acta - Bioenerg.* **1974**, *347* (1), 1–13. DOI: 10.1016/0005-2728(74)90194-7.

(10) Plumeré, N.; Nowaczyk, M. M. Biophotoelectrochemistry of Photosynthetic Proteins. In *Advances in biochemical engineering/biotechnology*; 2016; Vol. 123, pp 127–141. DOI: 10.1007/10_2016_7.

(11) Friebe, V. M.; Frese, R. N. Photosynthetic Reaction Center-Based Biophotovoltaics. *Curr. Opin. Electrochem.* **2017**, *5* (1), 126–134. DOI: 10.1016/j.coelec.2017.08.001.

(12) Friebe, V. M.; Delgado, J. D.; Swainsbury, D. J. K.; Gruber, J. M.; Chanaewa, A.; Van Grondelle, R.; Von Hauff, E.; Millo, D.; Jones, M. R.; Frese, R. N. Plasmon-Enhanced Photocurrent of Photosynthetic Pigment Proteins on Nanoporous Silver. *Adv. Funct. Mater.* **2016**, *26* (2), 285–292. DOI: 10.1002/adfm.201504020.

(13) Nawrocki, W. J.; Jones, M. R.; Frese, R. N.; Croce, R.; Friebe, V. M. In Situ Time-Resolved Spectroelectrochemistry Reveals Limitations of Biohybrid Photoelectrode Performance. *SSRN Electron. J.* **2022**. DOI: 10.2139/ssrn.4149955.
